# A Hopeless Case

**Published:** 1872-02

**Authors:** 


### A HOPELESS CASE.

The physician of experience often decides, before a visitor can take his seat, that it is a hopeless case; that he must die, or that he must live long, and can never be well again, which in many cases is a worse fate than to be laid in an early grave.

It is bad enough to be the victim of a living death, but to have two eventual fatalities gnawing at our vitals, is more than any mortal can bear with equanimity; hence we commiserate from the bottom of our heart the grave case of an eminent clerical friend of ours, who closes a long list of grievances with the culminating facts, "I have chronic diarrhœa, and — no salary."

